# Lupus érythémateux systémique à début pédiatrique: à propos d'un cas

**DOI:** 10.11604/pamj.2015.20.25.5769

**Published:** 2015-01-12

**Authors:** Sana Khlif, Hend Hachicha, Faten Frikha, Sawsan Feki, Mourad Ben Ayed, Zouhir Bahloul, Hatem Masmoudi

**Affiliations:** 1 Laboratoire d'Immunologie CHU Habib Bourguiba, 3029 Sfax, Tunisie; 2 Service de Médecine interne, CHU Hédi Chaker, 3029 Sfax, Tunisie

**Keywords:** Lupus érythémateux systémique, enfant, nourrisson, systemic lupus erythematosus, child, infant

## Abstract

Le lupus érythémateux systémique (LES) est une maladie systémique auto-immune d’étiologie inconnue qui touche essentiellement les femmes à l’âge adulte. Le lupus pédiatrique est une entité rare. Nous rapportons une nouvelle observation. Il s'agissait d'un nourrisson âgé de 7 mois qui présentait des lésions cutanées purpuriques, une polyarthrite fébrile. Le bilan immunologique était positif (AAN et anti-ADN). Une amélioration clinique et biologique a été notée sous corticothérapie générale avec une récidive lors de la dégression du traitement.

## Introduction

Le lupus érythémateux systémique (LES) à début pédiatrique est une entité rare, puisque seuls 10 à 15% des cas sont diagnostiqués avant l’âge de 16 ans [1, 2]. Il est caractérisé par sa plus grande sévérité par rapport au LES de l'adulte liée à la plus grande fréquence d'atteintes rénales, hématologiques et neurologiques sévères [3]. Il nécessite une corticothérapie dans la majorité des cas. Cette entité présente une morbidité importante à long terme. Dans ce travail, nous rapportons un cas de LES du nourrisson et nous faisons une revue de la littérature, avec discussion des particularités cliniques, diagnostiques et pathogéniques.

## Patient et observation

Il s'agit d'un nourrisson C.K, issu d'un mariage consanguin, né le 9 Aout 2011 d'un accouchement par césarienne suite à une souffrance fœtale aiguë. Sa croissance était normale (Poids=3.55 Kg, Taille=50 Cm, Périmètre Crânien=35 cm). Sa vaccination était normale. A l’âge de 7 mois, il présentait des lésions cutanées purpuriques localisées aux membres inférieurs. Le diagnostic retenu était celui d'un œdème aigu hémorragique (équivalent du purpura rhumatoïde chez le petit enfant). Trois mois après, soit à l’âge de 10 mois, sa mère constatait l'installation d'une polyarthrite touchant les 2 poignets et les articulations interphalangiennes des 2 mains. Un bilan biologique réalisé trouvait une Vitesse de sédimentation (VS) à 82 mm la 1^ère^ heure; et une protéine C-réactive (CRP) positive à 35,6 mg/l. En juin 2012, soit à l’âge de 11 mois, il était hospitalisé pour un purpura vasculaire des membres inférieurs évoluant depuis 3mois, une fièvre depuis 15 jours, une polyarthrite, le tout évoluant dans un contexte d'altération de l’état général (anorexie et amaigrissement) avec la notion de sueurs nocturnes. L'examen à l'admission trouvait un enfant fébrile, l'auscultation cardiopulmonaire et l'examen abdominal étaient normaux. L'examen cutané objectivait des lésions purpuriques diffuses des membres inférieurs, sans nécrose. A la biologie, la VS était à 110 mm à la première heure, la CRP était à 75 mg/l. L'hémogramme montrait une anémie d'allure inflammatoire à 7.1g /dl, une leuconeutropénie et une hyperlymphocytose à 8530 éléments/mm3 (76%), sans thrombopénie. Le Test de Coombs direct était négatif. La ferritinémie était à 160 ug /l. La créatinémie était à 42 umol/l. La calcémie était à 2.25umol/l. L’électrophorèse des protéines sériques était sans anomalies en particuliers absence d'hypergammaglobulinémie. Le bilan urinaire était normal. La radiographie thoracique était normale, ainsi que le myélogramme.

Un bilan immunologique complet a montré: des anticorps anti-nucléaires (AAN) positifs avec un aspect homogène et un taux ›1/1280, la recherche a été effectuée par technique d'immunofluorescence indirecte (IFI) sur cellules Hep2 (Biorad^®^), avec des anticorps anti-ADN natif positifs par IFI sur Crithidia Lucilae (Biosystems^®^) et des anticorps anti-nucléosomes fortement positifs par technique immunodot (ANA Profile 3 EUROLINE, EUROIMMUN^®^); Les anticorps anti-cardiolipines IgG -A- M recherchés par technique ELISA (Biomaghreb^®^) étaient franchement positifs (48RU/ml). Les IgG anti-beta2 glycoprotéine1 (ELISA Biomaghreb^®^) était positifs à 10U/ml; les anticorps anti-cytoplasme des polynucléaires neutrophiles (ANCA) étaient négatifs (technique d'IFI, EUROIMMUN^®^); le complément hémolytique total était effondré <10unités CH50/ml. Les fractions C3 (73 mg/dl) et C4 (5 mg/dl) du complément étaient diminuées; le dosage pondéral des immunoglobulines a montré des taux normaux avec des Ig G à 7.21 g/l des Ig A à 1.53 g/l et des Ig M à 0.38 g/l; un bilan immunologique de la mère était revenu négatif. L'immunophénotypage par cytométrie en flux, demandé devant l'hyperlymphocytose, montrait des populations lymphocytaires normales, absence de prolifération lymphoïde B monoclonale recherchée dans le cadre d'un lymphome. Le dosage pondéral des immunoglobulines a montré: Ig G= 7.21 g/l, Ig A= 1.53 g/l, Ig M= 0.38 g/l. Son typage HLA était le suivant: A10, A11, B52, B41, DRB1*15, DRB1*13, DRB5*, DRB3*. Les sérologies du virus d'Epstein Barr (EBV), des pneumopathies atypiques, des Bartonelles, des Rickettsioses, du CMV,étaient toutes négatives. L’échographie cardiaque, réalisée devant la suspicion d'un syndrome de Kawasaki pour la recherche d'anévrysmes coronariens, était normale. L’échographie abdominale n'avait pas montré d'anomalie décelable. Le diagnostic de LES à début pédiatrique selon les critères de l'ACR [4] (polyarthrite, leuconeutropénie, AAN fortement positif, Anti-ADN positif et ACL positifs) était alors retenu. L'enfant était traité par une corticothérapie à forte dose (Prednisone 60 mg/m^2^ par jour) avec une évolution favorable sur le plan clinique (disparition des signes généraux, des lésions cutanées et de la polyarthrite) et sur le plan biologique avec normalisation de la VS (23 mm à la 1^ère^ heure après un mois de traitement). A la dégression de la corticothérapie, on signalait l'apparition de lésions cutanées à type d’érythème polymorphe diffus aux deux membres inférieurs et à l'abdomen. L'examen par IFD d'une biopsie cutanée de ces lésions érythémato-papuleuses, révélait des dépôts vasculaires de C3. Ces lésions se sont améliorées spontanément. Une dose d'entretien de 10 mg par jour de prednisone était maintenue avec absence de rechute. Le recul actuel est de 32 mois.

## Discussion

Le terme de LES pédiatrique ou infantile a été utilisé pour décrire les enfants ayant présenté des manifestations cliniques ainsi qu'une sérologie positive du LES classique et dont les mères ont des anticorps anti-SSA, anti-SSB et anti-RNP négatifs [5]. Les cas décrits dans la littérature sont rares [6–10]. A la différence du LES néonatal, le LES pédiatrique peut présenter les manifestations cliniques et les complications du LES classique de l'adulte. Le taux de prévalence du LES pédiatrique est nettement inférieur à celui de l'adulte. Le taux d'incidence annuel du LES chez les enfants < 16 ans est inférieur à 1 pour 100.000 personnes dans les études d'Europe et d'Amérique du nord. A Taiwan, la prévalence du LES de l'enfant a été estimée à 6.3 pour 100.000 [2]. Le LES à début pédiatrique se différencie du LES de l'adulte par un sex-ratio fille/garçon moins élevé que chez l'adulte de 1/5 à 1/18 [11], comme chez notre nourrisson qui était de sexe masculin. Les éléments cliniques et biologiques du diagnostic et du suivi du LES pédiatrique sont identiques à ceux de l'adulte [4, 12]. Les manifestations initiales sont très variables avec des symptômes non spécifiques et trompeurs: fièvre, anorexie, perte de poids et asthénie. Au début de la maladie, un seul organe peut être atteint, mais la forme systémique est la forme de révélation habituelle. Les arthrites, l’éruption cutanée et l'atteinte rénale sont les atteintes les plus fréquentes de la forme pédiatrique [12]. Près de la moitié des enfants atteints de LES à l’âge pédiatrique possèdent des ACL ou un anticoagulant circulant, mais seul un faible nombre d'entre eux développe une maladie thrombotique, comme était le cas chez notre nourrisson [12]. Le bilan étiologique a permis de retenir le diagnostic de LES infantile après l'exclusion préalable des autres causes qui peuvent être évoquées même en association avec le lupus:

### Infections virales

#### *infection à PVB19

Des observations d'infections à PVB19 simulant un LES ont été rapportées sous forme de cas isolés ou de séries de patients. L’éruption due à PVB19 est accompagnée de polyarthrites et de polyarthralgies dans 60% des cas. Les AAN, les anticorps anti-ADNn et les antiphospholipides peuvent être positifs au cours de l'infection à PVB19 [13]. L'association de signes cutanés avec parfois un rash malaire, de fièvre, d'une arthropathie non destructrice, d'hypocomplémentémie et d'AAN de spécificités diverses a fait discuter le rôle du PVB19 dans la genèse du LES [14, 15]. De plus, plusieurs observations de LES survenues dans les suites d'une infection virale résolutive ont suggéré que le PVB19 pouvait être le facteur déclenchant d'une maladie systémique. Cependant, les études réalisées sur de grandes séries ont montré qu'en fait le PVB19 était une cause exceptionnelle de LES. De point de vue physiopathologique, le PVB19 interagirait avec les cellules inflammatoires par l'intermédiaire de la régulation de cytokines. Deux études récentes, suggèrent que l'infection virale par PVB19 pourrait modifier l'expression biologique du LES. Ces données préliminaires nécessitent une confirmation pour déterminer le rôle effectif du PVB19 au cours des maladies systémiques [13]. Vingt-huit observations d'infections à PVB19 associées au LES ont été rapportées dans la littérature. Les patients ne remplissant pas au moins trois des critères de l'ACR [4] pour le diagnostic du LES ont été exclus [16]. La prédominance féminine est nette. L’âge moyen est de 30,5 ans (6-71 ans).

*Les autres infections virales ont été éliminées par les sérologies virales.

### Lupus induit

Certains médicaments peuvent induire l'apparition d'un LES. Citons la minocycline, drogue fréquemment incriminée chez l'adolescent, l'acébutolol, la quinidine, la chlorpromazine, la carbamazépine, l'interféron α, et récemment, les agents anti-TNF [17]. Il est caractérisé par une moindre prédominance féminine, une fréquence élevée de manifestations générales et de pleuropéricardite et la rareté des atteintes rénales sévères. Biologiquement, il est distingué par la présence de titres élevés d'anticorps anti-histones. Chez notre patient, il n'y avait pas de notion de prise médicamenteuse d'autant plus que les anticorps anti-histones étaient négatifs.

### Maladie de Kawasaki

C'est une maladie pédiatrique qui atteint les enfants avant l’âge de 5 ans avec un pic de fréquence entre 1 et 2 ans. Bien qu'elle soit fréquente au Japon, elle est décrite dans le monde entier. Son diagnostic est essentiellement clinique reposant sur 6 critères principaux (fièvre supérieure à 5 jours, rash tronculaire, adénopathies cervicales, modifications des extrémités, atteinte de la cavité buccopharyngée, injection conjonctivale bilatérale). En effet, l'atteinte de nombreux organes de l'organisme fait souvent évoquer une maladie infectieuse. Sa gravité tient à la survenue de complications cardiaques qui sont les thromboses et les anévrismes en particulier coronaires [18].

### Déficit héréditaire du complément

Il s'agit d'une situation particulière dans laquelle un déficit homozygote affectant un gène unique est associé à la survenue d'un LES avec un début pédiatrique. Ce déficit affecte les gènes impliquées à la phase initiale d'activation de la voie classique du complément, tels C1q (90% de LES en cas de déficit complet), C1r, C1s ou C4 [19]. Le LES est une maladie auto-immune dont la physiopathologie est multifactorielle résultant de l'interaction de facteurs environnementaux, immunologiques et génétiques [3]. C'est une entité hétérogène sur le plan génétique, comportant des formes polygéniques (particulièrement dans les formes à début adulte) et des formes monogéniques associées à la mutation d'un seul gène (particulièrement dans les formes à début pédiatrique). Au cours des dix dernières années, l’étude des formes précoces, familiales ou syndromiques ont permis la description de nouvelles causes monogéniques de lupus. L'identification en 2011 de deux entités de transmission mendélienne associées au développement d'un LES pédiatrique est en faveur de l'hypothèse d'une pathologie monogénique [3]. On peut raisonnablement faire l'hypothèse que la survenue d'un LES chez notre nourrisson serait monogénique surtout devant la notion de consanguinité chez les parents. La prise en charge et la prévention des poussées sont établies selon les mêmes recommandations que chez l'adulte [11]. La corticothérapie est souvent nécessaire chez l'enfant et doit être utilisée à faible dose dans les formes peu sévères, et précocement à fortes doses dans les formes graves. La prescription d'hydroxychloroquine doit être systématique. La prévention des complications repose comme chez l'adulte sur les mesures de photoprotection et la prévention des infections. Notre nourrisson était traité par corticothérapie à forte dose et dégressive jusqu’à une dose d'entretien de 10 mg/jour. Nous n'avons pas eu recours systématique à l'hydroxychloroquine dont les conséquences oculaires ne sont pas bien connues à long terne si pris précocement chez ces malades.

## Conclusion

Le LES pédiatrique, plus sévère que la forme adulte, doit être reconnu et traité précocement. La morbidité et la mortalité dépendent des systèmes d'organes touchés. Les enfants atteints ont un excellent pronostic à long terme lorsque les lésions cutanées seules sont présentes. La sévérité du lupus pédiatrique impose un diagnostic et un traitement précoce afin d'assurer le meilleur contrôle de l'inflammation et d’éviter la morbi-mortalité associée. Ce traitement repose sur une prise en charge pluridisciplinaire afin de relever les défis d'une croissance staturale satisfaisante de l'enfant.
